# Effectiveness of probiotic in preventing and treating antibiotic-associated diarrhoea and/or *Clostridium difficile*-associated diarrhoea in patients with spinal cord injury: a protocol of systematic review of randomised controlled trials

**DOI:** 10.1186/s13643-015-0159-3

**Published:** 2015-11-24

**Authors:** Samford Wong, Ali Jamous, Jean O’Driscoll, Ravi Sekhar, Mofid Saif, Steve O’Driscoll, Sarah Lewis, Eamonn McKeown, Shashi P. Hirani

**Affiliations:** National Spinal Injuries Centre, Stoke Mandeville Hospital, Aylesbury, HP21 8AL UK; Centre for Health Services Research, City University, London, UK; Institute for Liver and Digestive Health, University College London, London, UK; Royal Buckinghamshire Hospital, Aylesbury, UK; Department of Microbiology, Stoke Mandeville Hospital, Aylesbury, UK; Department of Gastroenterology, Stoke Mandeville Hospital, Aylesbury, UK; Library Services, Stoke Mandeville Hospital, Aylesbury, UK

**Keywords:** Spinal cord injury centres, Probiotics, *Clostridium difficile*, Antibiotic-associated diarrhoea

## Abstract

**Background:**

Probiotics may prevent antibiotic-associated and *Clostridium difficile*-associated diarrhoea (AAD/CDAD). Many spinal cord injury centre (SCIC) practitioners consider probiotics generically and may not realise that efficacy can be strain-, dose- and disease-specific. In order to confirm these effects and fully evaluate the extent of probiotic effectiveness in these patients, a systematic review and meta-analysis is indicated.

**Methods:**

The following databases will be searched for relevant studies: Cochrane Library; Centre for Reviews and Dissemination (CRD) Database; CINAHL; PsycINFO; Embase; Medline; AMED; International Clinical Trials Registry Platform Search Portal and ISRCTN Registry and will hand search a list of conference proceedings. Any randomised controlled trials without restriction of publication status will be included with treatment of AAD/CDAD. Outcomes will include the effect of probiotic on the occurrence of AAD/CDAD and duration of diarrhoea, intensive care unit admission, hospital mortality and length of hospital stay. Two reviewers will independently screen the titles, abstracts or even full texts and extract data. Two other reviewers will assess study quality. Revman 5.1 software will be used to conduct meta-analysis and calculate the risk ratio for dichotomous data. Weighted mean difference or standard mean difference will be calculated for continuous data. The Cochrane Collaboration’s tool will be used to assess the risk of bias.

**Discussion:**

This systematic review protocol will provide information on probiotic therapy for AAD and CDAD in spinal cord injury (SCI) population. The results will be disseminated through peer-reviewed publication or conference presentation.

**Systematic review registration:**

PROSPERO CRD42015016976

**Electronic supplementary material:**

The online version of this article (doi:10.1186/s13643-015-0159-3) contains supplementary material, which is available to authorized users.

## Background

### Introduction

Data on the prevalence of diarrhoea associated with antibiotic used (AAD) and *Clostridium difficile* (CDAD) in spinal cord injury (SCI) patients is limited. Recent meta-analysis examining randomised, double-blinded, controlled trials of probiotics in the prevention of AAD [1–4] and CDAD [4] suggest the use of probiotic is associated with a reduction in diarrhoea compared to placebo. However, the heterogeneity of the studies (varies in study population, sample size, probiotic strains, dose and duration of probiotic and definition of diarrhoea) makes it very difficult to draw definite conclusions. Many physicians and consumers view all probiotics as being the same and therefore could apply to all populations. SCI are life-changing events that have significant medical, physical, socio-psychological effects. After SCI, patients are required to stay in SCI centres for an extended period of time before they can re-integrate back into community. Indeed, SCI patients are more vulnerable in developing AAD/CDAD as they tend to stay in hospitals for an extended period of time after an SCI and the use of antibiotics are common. We are not aware of any published systematic review reporting the effectiveness of probiotics in preventing or treating diarrhoea in SCI patients. It appeared logical to assess probiotics in SCI patients because these patients are particularly vulnerable to diarrhoea and its consequences for many reasons, such as the increased use of antibiotics for treating urinary tract infection due to increased catheter use [5]. Diarrhoea can delay rehabilitation, increase the risk of developing pressure ulcers/delay wound healing and reduce quality of life [5].

### Description of the intervention

Probiotics are live organisms that, when administered in optimum amounts, confer a health benefit on the host [6]. They are increasingly available as capsules and dairy-based food products sold in supermarkets and health food shops. Although there are numerous commercially available probiotics, there is much debate as to what beneficial effects these provide and which specific organisms may be most effective in any specific patient group [3, 7, 8]. Microorganisms commonly used in probiotic preparations include bacteria of the genera *Lactobacillus*, *Bifidobacterium*, *Escherichia*, *Enterococcus* or *Bacillus* and the fungal genus *Saccharomyces* [8].

### How the intervention might work

Probiotics that colonise the gastrointestinal tract (GI) effectively help resist gut colonisation by potentially harmful bacteria. Such probiotics often have additional properties that benefit the host [8, 9]. Certain *Lactobacillus* strains can produce antimicrobial compounds, known as bacteriocins, which may inhibit pathogens such as *Bacillus*, *Staphylococcus* and *Enterococcus* species. A specific strain of *Lactobacillus acidophilus* produces a bacteriocin that has shown to inhibit strains of *Listeria innocua* and *Listeria monocytogenes* [7].

### Why it is important to perform this review

Different probiotic species and strains can have substantially different effects on the host [9, 10]. Several species- and strain-specific factors play a role in determining what benefits, if any, a probiotic may confer. To exert a beneficial effect, a probiotic must first be able to colonise the GI tract. The initial step required for GI colonisation by probiotics is adhesion to the GI mucosa [11]. Although not fully understood, current evidence suggests that the adhesive characteristics of probiotics may be due to differences in the expression of large surface proteins and their interaction with mucus-binding proteins [11]. Probiotics have been suggested as a means of preventing adverse GI conditions such as antibiotic-associated diarrhoea (AAD) and *Clostridium difficile*-associated diarrhoea (CDAD) [3, 7, 10, 12]. However, this is not a characteristic that is shared amongst all probiotic strains [8–11], and effects may differ with different patient groups [9, 12]. One example where the use of probiotics is particularly likely to be beneficial is in patients with SCI who not only require an extended period of stay in hospital but also have increased risk of infection due to the use of urinary catheters for long-term bladder management. If diarrhoea develops, rehabilitation will be delayed, impacting not just on the patient but also causing significant extra healthcare costs. Given the severe loss of quality of life for SCI patients, if any probiotic is effective, their low cost as well as the low incidence of adverse events [6] render probiotics an attractive intervention to prevent AAD/CDAD.

Anecdotally, it has been noted that many practitioners consider probiotics in generic terms, not recognising that there may be differences between different products. Similarly, some of the healthcare facilities stock a probiotic but will not substitute one commercial probiotic for another based on cost or availability, and without regard for any scientific evidence to support the probiotic in question.

With this in mind, it is important to recognise that there is no ‘generic equivalency’ between probiotic species and strains. In clinical practice, it is important for clinicians to use or recommend specific commercially available probiotics that have specifically been shown to have beneficial effects in clinical trials.

To address this issue, we plan to conduct a systematic review and meta-analysis to determine: (1) if probiotic is effective in preventing or treating diarrhoea associated with antibiotic use and *Clostridium difficile* infection; (2) what is the optimal dose, duration and frequency of probiotics in SCI patients.

## Methods and analysis

### Eligibility criteria for included studies

#### Type of studies

Randomised Controlled Trials (RCTs) in English will be included without restriction of publication type.

#### Participants

Participants aged 18 years and over, any race or gender with a diagnosis of spinal cord injury (according to the definition of the International Standards for neurological classification of spinal cord injury and American Spinal Injury Association (ASIA) Impairment Scale (AIS) A-D [13]) will be eligible for the systematic review and meta-analysis.

#### Type of intervention

Probiotic administration (all strains and dose information will be recorded) in the intervention group must be given within 5 days of antibiotic commencement. The control group should receive either placebo or routine clinical care. The reason we would like to ensure the study administered probiotics within 5 days of antibiotic commencement is due to minimising the risk of dysbiosis [14].

#### Study end points/main outcomes

The primary study end points include the incidence of diarrhoea associated with antibiotic use and *Clostridium difficile* infection. The definition of diarrhoea and occurrence of AAD/CDAD and its follow-up period will be recorded as per identified paper.

The secondary end points include duration of diarrhoea, Intensive Care Unit (ICU) admission, hospital mortality, length of hospital stay and occurrence of adverse events.

### Search methods for identifying studies

#### Electronic searches

We will systematically search Cochrane Library, Centre for Reviews and Dissemination (CRD), CINAHL, PsycINFO, Embase, Medline and AMED from inception to 27th February 2015. We will also screen the reference lists of relevant studies and reviews for additional articles. In addition, we will search the following websites for unpublished or ongoing studies: International Clinical Trial Registry Platform Search Portal (http://www.who.int/ictrp/search/en/) and ISTCTN registry (http://www.controlled-trials.com) and review abstracts from selected scientific proceedings (*Proceeding of the Nutrition Society* of the Nutrition Society of the UK and *Clinical Nutrition Supplement* or *e-ESPEN* of the European Society of Parenteral and Enteral Nutrition). We will apply a language filter in this study. Studies reported in non-English language will be excluded.

#### Search terms/search strategy

The keywords and Medical Subject Headings related to probiotic (lactobacillus, bifidobacter*, bifidobacillus, streptococc*, lactococc*, leuconostoc, pediococc*, saccharomyce, probiotic and synbiotic), diarrhoea (antibiotic associated, *Clostridium difficile* associated) and SCI patients (spine injury, cervical injury, spine fracture, vertebra compression, vertebra dislocation, quadriplegia, paralysis, paraplegia, tetraplegia, paraplegia, thoracic injury, lumbar injury, sacral injury) will be used alone or in combination (and with synonyms and closely related words) to retrieve relevant articles. The search strategies have been developed (see Additional file 1 for Medline/Embase/CINAHL/AMED/PsycINFO/Cochrane/Centre for Reviews and Dissemination/International Clinical Trials Registry), and a similar search strategy will be adapted for the other databases.

### Data collection and analysis

**Fig. 1:**
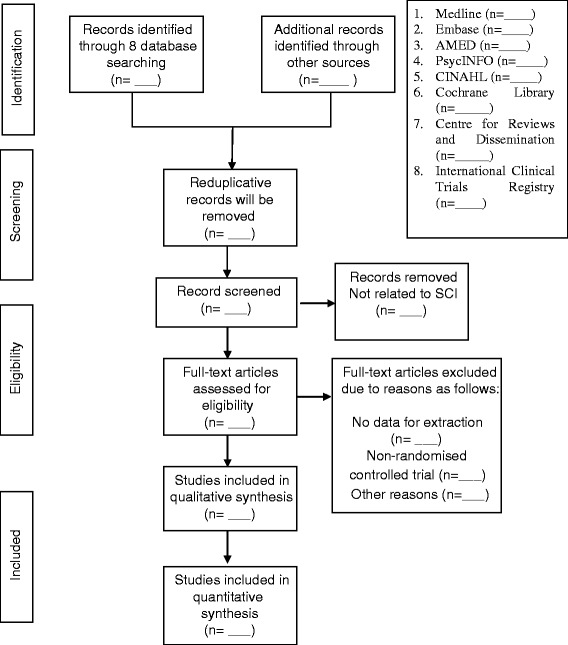
Process of the systematic review

Three reviewers (SW, SH and EM) will independently examine the titles and/or abstracts and eliminate irrelevant studies. The full text of all potential eligible studies will be read and their suitability for inclusion determined according to the PICO (participant, intervention, comparison and outcomes) model. Discrepancy will be resolved by consensus or discussion with other reviewers (AJ, JO’D). Inter-rater agreement will be assessed using the kappa statistics (*Κ* < 0.01: no agreement; *Κ*: poor agreement; *Κ* = 0.21–0.4: fair agreement; *Κ* = 0.41–0.6: moderate agreement; *Κ* = 0.61–0.8: good agreement; *Κ* = 0.81–1: very good agreement) [15]. Details of the study selection procedure are shown in Fig. 1. Excluded studies will be listed in a table with the reasons for exclusion outlined.

#### Data extraction

All abstract data extracted from the retrieved trials will be reviewed independently using a predefined data extraction sheet. Any discrepancy will be managed by consensus. The following variables will be recorded for each study: the name of the first author, publication year, country of origin, type of setting (SCI centres, general hospital, community), patients’ characteristics (gender, age, number, inclusion and exclusion criteria), characteristics of interventions (type of probiotics, concentration, route, dose and duration of intervention), characteristics of control methods, and outcomes (occurrence of diarrhoea, mortality, ICU admission, the length of hospital stay and adverse event data, in two groups). If necessary (unclear data, missing data and extractable data), we will attempt to contact the corresponding authors of the included studies for missing data and for clarification.

#### Risk of bias assessment

The reviewers will independently assess the risk of bias using the assessment tool from the Cochrane Collaboration [16]. The following sources of bias will be detected: selection bias (random sequence generation and allocation concealment), detection bias (blinding of outcome assessment), blinding of participants and personnel (performance bias), attrition bias (incomplete outcome data), reporting bias (selective outcome reporting) and industry bias. The studies will then be classified into three levels of bias: low, unclear and high risk bias. Differences in opinion will be resolved by discussion or consultation with third reviewer (EM).

#### Assessment of the quality of included studies

The quality of the evidence will be rated according to the GRADE guidelines [17, 18].

#### Dealing with missing data

If there is any missing or insufficient data in included studies, we will contact the corresponding authors of the study by email or telephone to obtain more information. If we are unable to obtain the missing data, the method reported by the Cochrane Handbook for Systematic Reviews of Intervention [16] will be used to perform complete case analysis, and a sensitivity analysis will also be conducted to assess the impact of including studies with 20 % or more of missing data. For all outcomes, we will conduct intention-to-treat analysis wherever possible.

#### Statistical analysis

Revman (version 5.1) software will be used to conduct a meta-analysis and calculate the OR and 95 % confidence interval (CI) for dichotomous data. Weighted mean difference (WMD) or standard mean difference (SMD) and 95 % CI will be calculated for continuous data. If the same outcome measurement tool and unit was used, the WMD and 95 % CI will be calculated, or otherwise the SMD and 95 % CI. To account for differences between probiotic interventions (different strains and dose), sub-group analyses by intervention will be conducted (if we identified more than three studies using similar probiotic strains). If the intervention are too varied, data will not be pooled.

Given the heterogeneity of study designs, probiotic strains and dosing regimens, a conservative approach will be employed for all analyses based on a random effect model. The RR, WMD or SMD will be calculated by the random-effect model. Forest plot will be generated to illustrate the study-specific effect sizes along with a 95 % CI.

#### Assessment of heterogeneity

Heterogeneity will be assessed using Cochran’s *Q* statistic and Higgins *I*^2^ statistic, where *I*^2^ > 50 % indicates the presence of significant heterogeneity. *I*^2^ will be calculated according to the equation *I*^2^ = 100 % × (*Q*-df)/*Q*, where *Q* is the Cochran heterogeneity statistic [19].

#### Sensitivity analysis

The sensitivity analysis will be used to assess whether the sample size and missing data impact on the results of the review. If there are adequate studies (not less than three studies), we will conduct a sensitivity analysis to check the robustness of conclusions and assess the impact of methodological quality.

For sensitivity analyses, we will perform meta-analyses using fixed effects models and assess the consistency of our results across random-effect models and fixed effects models.

#### Assessment of publication bias

A funnel plot will be used to evaluate publication bias if more than ten studies are included. A symmetrical funnel plot usually suggests an absence of publication bias. However, asymmetry in a funnel plot can be explained by other factors including publication bias and differing study quality [20]. We will also use Egger’s test [21] to qualitatively detect publication bias.

#### Ethics and dissemination

Ethics approval is not required as this is a protocol for a systematic review. The findings will be disseminated in a peer-reviewed journal and presented at a relevant conference. The study is registered at PROSPERO, the International Prospective Register of Systematic Reviews, at the University of York (CRD 42015016976).

## Discussion

This systematic review and meta-analysis will use the most definitive method to assess the effectiveness of probiotic in preventing and treating diarrhoea associated with antibiotics and *Clostridium difficile* infection in SCI population.

The current use of probiotic to prevent and treat AAD/CDAD remains inconclusive, where it could be limited by variation in strain, dose, duration and disease-type studied. This systematic review and meta-analysis will examine the potential impact of probiotic intervention in preventing or treating AAD/CDAD in SCI patients. Other specific aims that will be addressed by this study include determining the strains, optimal dose and duration of probiotic and the safety profile of probiotics. If probiotics are found to be effective, this may support the need for routine prescriptions to SCI patients as prophylactic or to treat AAD/CDAD. The collaboration formed through this study will be the platform to conduct further a systematic review and meta-analysis for the probiotic management and prevention of diarrhoea in SCI patients.

## Additional file

Additional file 1:
**The search strategies for Cochrane Library / Centre for Reviews and Dissemination database /CINAHL/ PsycINFO/ Embase/ Medline/ AMED.**

## Abbreviations

AAD: antibiotic-associated diarrhoea
AIS: ASIA Impairment Scale
ASIA: American Spinal Injury Association
CDAD: *Clostridium difficile*-associated diarrhoea
GI: gastrointestinal tract
ICU: intensive care unit
SCI: spinal cord injury
SCIC: spinal cord injury centre
