# Supplementary material for: Effectiveness of probiotic in preventing and treating antibiotic-associated diarrhoea and/or Clostridium difficile-associated diarrhoea in patients with spinal cord injury: a protocol of systematic review of randomised controlled trials
Source: Syst Rev. 2015 Nov 24;4:170. doi: 10.1186/s13643-015-0159-3 (PMC4657267; doi:10.1186/s13643-015-0159-3)
Supplement: Additional file 1: — The search strategies for Cochrane Library / Centre for Reviews and Dissemination database /CINAHL/ PsycINFO/ Embase/ Medline/ AMED. [file 13643_2015_159_MOESM1_ESM.docx]

Supplementary file: search strategy

**Database: Ovid MEDLINE(R) <1946 to 23 February 2015>**

Search Strategy:

--------------------------------------------------------------------------------

1 exp probiotic agent/

2 "probio*".ti,ab.

3 "synbio*".ti,ab.

4 exp synbiotic agent/

5 acidophilus.ti,ab.

6 delbruecki.ti,ab.

7 johnsonii.ti,ab.

8 plantarum.ti,ab.

9 fermentum.ti,ab.

10 reuteri.ti,ab.

11 rhamnosus.ti,ab.

12 salivarius.ti,ab.

13 bifidum.ti,ab.

14 "bifidobacteri*".ti,ab.

15 exp bifidobacterium/ or bifidobacteriaceae/

16 saccharomycetales/ or exp saccharomyces/

17 "saccharomyc*".ti,ab.

18 enterococcaceae/ or exp enterococcus/

19 "enterococc*".ti,ab.

20 "dairy product* ".ti,ab.

21 exp yoghurt/

22 "yoghurt*".ti,ab.

23 "fermented product* ".ti,ab.

24 kefir.ti,ab.

25 bacillales/

26 lactobacillales/ or exp lactobacillus/ or lactobacillaceae/

27 "lactobacill*".ti,ab.

28 (casei or paracasei).ti,ab.

29 shirota.ti,ab.

30 ("Lactobac* casei Shirota" or LcS).ti,ab.

31 (lactobac* adj3 shirota).mp. [mp=title, abstract, original title, name of substance word, subject heading word, keyword heading word, protocol supplementary concept word, rare disease supplementary concept word, unique identifier]

32 amylovorus.ti,ab.

33 bifidus.ti,ab.

34 brevis.ti,ab.

35 buchneri.ti,ab.

36 bulgaricus.ti,ab.

37 crispatus.ti,ab.

38 curvatus.ti,ab.

39 delbrueckii.ti,ab.

40 gasseri.ti,ab.

41 sakei.ti,ab.

42 pentosus.ti,ab.

43 helveticus.ti,ab.

44 "streptococc*".ti,ab.

45 streptococcaceae/ or exp streptococcus/

46 exp Probiotics/

47 exp Synbiotics/

48 1 or 2 or 3 or 4 or 5 or 6 or 7 or 8 or 9 or 10 or 11 or 12 or 13 or 14 or 15 or 16 or 17 or 18 or 19 or 20 or 21 or 22 or 23 or 24 or 25 or 26 or 27 or 28 or 29 or 30 or 31 or 32 or 33 or 34 or 35 or 36 or 37 or 38 or 39 or 40 or 41 or 42 or 43 or 44 or 45 or 46 or 47

49 exp Diarrhea/

50 "diarrh*ea*".ab,ti.

51 "c*diff* ".ab,ti.

52 (clostridium adj2 diff*).mp. [mp=title, abstract, original title, name of substance word, subject heading word, keyword heading word, protocol supplementary concept word, rare disease supplementary concept word, unique identifier]

53 exp Clostridium/ or exp Clostridium difficile/ or exp Clostridium Infections/ or exp Enterocolitis, Pseudomembranous/

54 (AAD or CDAD).ab,ti.

55 exp Gastroenteritis/

56 "gastroenter*".ab,ti.

57 exp Anti-Bacterial Agents/ae [Adverse Effects]

58 49 or 50 or 51 or 52 or 53 or 54 or 55 or 56 or 57

59 48 and 58

60 exp Spinal Cord Injuries/

61 exp Spinal Injuries/ or exp Cervical Vertebrae/ or exp Spinal Fractures/ (44358)

62 SCI.ab,ti.

63 ((spinal or spine or cervical or thoracic or lumbar or sacral) adj3 (trauma* or injur* or contus* or lacerat* or transection* or lesion* or wound* or broken or break* or fractur* or damag*)).mp. [mp=title, abstract, original title, name of substance word, subject heading word, keyword heading word, protocol supplementary concept word, rare disease supplementary concept word, unique identifier]

64 ((spinal or spine or cervical or thoracic or lumbar or sacral) adj3 (trauma* or injur* or contus* or lacerat* or transection* or lesion* or wound* or broken or break* or fractur* or damag*)).ab,ti.

65 ("central cord" adj3 (trauma* or injur* or contus* or lacerat* or transection* or lesion* or wound* or broken or break* or fractur* or damag*)).ab,ti.

66 exp Paraplegia/

67 "parapleg*".ab,ti.

68 paraparesis.ab,ti.

69 "quadrapleg*".ab,ti.

70 quadraparesis.ab,ti.

71 "quadripleg*".ab,ti.

72 quadriparesis.ab,ti.

73 exp Quadriplegia/

74 "tetrapleg*".ab,ti.

75 tetraparesis.ab,ti.

76 (spine or spinal).jw.

77 ("spinal cord" adj3 patient*).mp. [mp=title, abstract, original title, name of substance word, subject heading word, keyword heading word, protocol supplementary concept word, rare disease supplementary concept word, unique identifier]

78 60 or 61 or 62 or 64 or 65 or 66 or 67 or 68 or 69 or 70 or 71 or 72 or 73 or 74 or 75 or 76 or 77

79 59 and 78 ***************************

**Database: Embase <1980 to 23 February 2015>**

Search Strategy:

--------------------------------------------------------------------------------

1 exp probiotic agent/

2 "probio*".ti,ab.

3 "synbio*".ti,ab.

4 exp synbiotic agent/

5 acidophilus.ti,ab.

6 delbruecki.ti,ab.

7 johnsonii.ti,ab.

8 plantarum.ti,ab.

9 fermentum.ti,ab.

10 reuteri.ti,ab.

11 rhamnosus.ti,ab.

12 salivarius.ti,ab.

13 bifidum.ti,ab.

14 "bifidobacteri*".ti,ab.

15 exp bifidobacterium/ or bifidobacteriaceae/

16 saccharomycetales/ or exp saccharomyces/

17 "saccharomyc*".ti,ab.

18 enterococcaceae/ or exp enterococcus/

19 "enterococc*".ti,ab.

20 "dairy product* ".ti,ab.

21 exp yoghurt/

22 "yoghurt*".ti,ab.

23 "fermented product* ".ti,ab.

24 kefir.ti,ab.

25 bacillales/

26 lactobacillales/ or exp lactobacillus/ or lactobacillaceae/

27 "lactobacill*".ti,ab.

28 (casei or paracasei).ti,ab.

29 shirota.ti,ab.

30 ("Lactobac* casei Shirota" or LcS).ti,ab.

31 (lactobac* adj3 shirota).mp. [mp=title, abstract, subject headings, heading word, drug trade name, original title, device manufacturer, drug manufacturer, device trade name, keyword] (203)

32 amylovorus.ti,ab.

33 bifidus.ti,ab.

34 brevis.ti,ab.

35 buchneri.ti,ab.

36 bulgaricus.ti,ab.

37 crispatus.ti,ab.

38 curvatus.ti,ab.

39 delbrueckii.ti,ab.

40 gasseri.ti,ab.

41 sakei.ti,ab.

42 pentosus.ti,ab.

43 helveticus.ti,ab.

44 "streptococc*".ti,ab.

45 streptococcaceae/ or exp streptococcus/

46 1 or 2 or 3 or 4 or 5 or 6 or 7 or 8 or 9 or 10 or 11 or 12 or 13 or 14 or 15 or 16 or 17 or 18 or 19 or 20 or 21 or 22 or 23 or 24 or 25 or 26 or 27 or 28 or 29 or 30 or 31 or 32 or 33 or 34 or 35 or 36 or 37 or 38 or 39 or 40 or 41 or 42 or 43 or 44 or 45

47 exp spinal cord injury/

48 exp cervical spine injury/

49 exp spine injury/

50 exp cervical spinal cord injury/

51 SCI.ti,ab.

52 ((spine or spinal or cervical or thoracic or lumbar or sacral) adj3 (trauma* or injur* or contus* or lacerat* or transection* or lesion* or wound* or broken or break* or fractur* or damag*)).mp. [mp=title, abstract, subject headings, heading word, drug trade name, original title, device manufacturer, drug manufacturer, device trade name, keyword]

53 ("central cord" adj3 (trauma* or injur* or contus* or lacerat* or transection* or lesion* or wound* or broken or break* or fractur* or damag*)).mp. [mp=title, abstract, subject headings, heading word, drug trade name, original title, device manufacturer, drug manufacturer, device trade name, keyword]

54 exp paraplegia/

55 (parapleg* or paraparesis).ti,ab.

56 exp quadriplegia/

57 (quadripleg* or quadriparesis).ti,ab.

58 (quadrapleg* or quadraparesis).ti,ab.

59 (tetrapleg* or tetraparesis).ti,ab.

60 ("spinal cord" adj3 patient*).mp. [mp=title, abstract, subject headings, heading word, drug trade name, original title, device manufacturer, drug manufacturer, device trade name, keyword]

61 (spine or spinal).jx.

62 47 or 48 or 49 or 50 or 51 or 52 or 53 or 54 or 55 or 56 or 57 or 58 or 59 or 60 or 61

63 46 and 62

64 exp diarrhea/

65 exp Clostridium difficile/

66 clostridium difficile/ or exp clostridium/ or exp clostridium difficile infection/

67 exp pseudomembranous colitis/

68 "diarrh*ea".ti,ab.

69 (AAD or CDAD).ti,ab.

70 "c*diff* ".ti,ab.

71 (clostridium adj2 diff*).mp. [mp=title, abstract, subject headings, heading word, drug trade name, original title, device manufacturer, drug manufacturer, device trade name, keyword]

72 exp gastroenteritis/

73 "gastroenter*".ti,ab.

74 exp antibiotic agent/ae [Adverse Drug Reaction]

75 64 or 65 or 66 or 67 or 68 or 69 or 70 or 71 or 72 or 73 or 74

76 63 and 75

**************************

**CINAHL Search strategy: 27 Feb 2015**

1. CINAHL; exp PROBIOTICS/;

2. CINAHL; probio*.ti,ab;

3. CINAHL; synbio*.ti,ab;

4. CINAHL; exp BIFIDOBACTERIUM/;

5. CINAHL; BACILLACEAE/;

6. CINAHL; bifidobacteri*.ti,ab;

7. CINAHL; saccharomyc*.ti,ab;

8. CINAHL; ENTEROCOCCUS/;

9. CINAHL; enterococc*.ti,ab;

10. CINAHL; streptococc*.ti,ab;

11. CINAHL; exp STREPTOCOCCUS/;

12. CINAHL; bifidum.ti,ab;

13. CINAHL; LACTOBACILLUS/ OR LACTOBACILLUS ACIDOPHILUS/;

14. CINAHL; lactobacill*.ti,ab;

15. CINAHL; acidophilus.ti,ab;

16. CINAHL; delbrueck*.ti,ab;

17. CINAHL; johnsonii.ti,ab;

18. CINAHL; plantarum.ti,ab;

19. CINAHL; fermentum.ti,ab;

20. CINAHL; reuteri.ti,ab;

21. CINAHL; rhamnosus.ti,ab;

22. CINAHL; salivarius.ti,ab;

23. CINAHL; amylovorus.ti,ab;

24. CINAHL; bifidus.ti,ab;

25. CINAHL; brevis.ti,ab;

26. CINAHL; buchneri.ti,ab;

27. CINAHL; bulgaricus.ti,ab;

28. CINAHL; crispatus.ti,ab;

29. CINAHL; curvatus.ti,ab;

30. CINAHL; gasseri.ti,ab;

31. CINAHL; pentosus.ti,ab;

32. CINAHL; helveticus.ti,ab;

35. CINAHL; 15 OR 16 OR 17 OR 18 OR 19 OR 20 OR 21 OR 22 OR 23 OR 24 OR 25 OR 26 OR 27 OR 28 OR 29 OR 30 OR 31 OR 32;

36. CINAHL; (casei OR paracasei).ti,ab;

37. CINAHL; shirota.ti,ab;

38. CINAHL; ("lactobac* caseishirota" OR LcS).ti,ab;

39. CINAHL; (lactobac* ADJ3 shirota).ti,ab;

40. CINAHL; "dairy product*".ti,ab;

41. CINAHL; "fermented product".ti,ab;

42. CINAHL; yoghurt*.ti,ab;

43. CINAHL; exp YOGURT/;

44. CINAHL; kefir.ti,ab;

45. CINAHL; 1 OR 2 OR 3 OR 4 OR 5 OR 6 OR 7 OR 8 OR 9 OR 10 OR 11 OR 12 OR 13 OR 14 OR 15 OR 16 OR 17 OR 18 OR 19 OR 20 OR 21 OR 22 OR 23 OR 24 OR 25 OR 26 OR 27 OR 28 OR 29 OR 30 OR 31 OR 32 OR 35 OR 36 OR 37 OR 38 OR 39 OR 40 OR 41 OR 42 OR 43 OR 44;

46. CINAHL; exp DIARRHEA/;

47. CINAHL; diarrh*ea*.ti,ab;

48. CINAHL; c*diff*.ti,ab;

49. CINAHL; (clostridium adj2 diff*).ti,ab;

51. CINAHL; exp CLOSTRIDIUM/ OR exp CLOSTRIDIUM INFECTIONS/ OR exp CLOSTRIDIUM DIFFICILE/

OR exp ENTEROCOLITIS, PSEUDOMEMBRANOUS/;

52. CINAHL; (AAD OR CDAD).ti,ab;

53. CINAHL; exp GASTROENTERITIS/;

54. CINAHL; gastroenter*.ti,ab;

55. CINAHL; exp ANTIBIOTICS/AE [AE=Adverse Effects];

56. CINAHL; 46 OR 47 OR 48 OR 49 OR 51 OR 52 OR 53 OR 54 OR 55;

57. CINAHL; 45 AND 56;

58. CINAHL; SPINAL CORD/ OR exp SPINAL CORD INJURIES/ OR exp SPINAL CORD INJURY NURSING/ OR exp SPINAL CORD COMPRESSION/;

59. CINAHL; exp SPINAL FRACTURES/;

60. CINAHL; exp SPINAL INJURIES/;

61. CINAHL; (cervical AND spine).ti,ab;

62. CINAHL; exp CERVICAL VERTEBRAE/;

63. CINAHL; ((spine OR spinal OR cervical OR thoracic OR lumbar OR sacral) adj3 (trauma* OR injur* OR contus* OR lacerat* OR transection* OR lesion* OR wound* OR broken OR break* OR fractur* OR wound* OR damag*)).ti,ab;

64. CINAHL; ("central cord" adj3 (trauma* OR injur* OR contus* OR lacerat* OR transection* OR lesion* OR wound* OR broken OR break* OR fractur* OR wound* OR damag*)).ti,ab; 28 results.

65. CINAHL; exp PARAPLEGIA/;

66. CINAHL; (parapleg* OR paraparesis).ti,ab;

67. CINAHL; exp QUADRIPLEGIA/;

68. CINAHL; quadripleg*.ti,ab;

69. CINAHL; quadriparesis.ti,ab;

70. CINAHL; quadrapleg*.ti,ab;

71. CINAHL; quadraparesis.ti,ab;

72. CINAHL; tetrapleg*.ti,ab;

73. CINAHL; tetraparesis.ti,ab;

74. CINAHL; (spine OR spinal).jn;

75. CINAHL; ("spinal cord" adj3 patient*).ti,ab;

76. CINAHL; 58 OR 59 OR 60 OR 61 OR 62 OR 63 OR 64 OR 65 OR 66 OR 67 OR 68 OR 69 OR 70 OR 71 OR 72 OR 73 OR 74 OR 75;

77. CINAHL; 57 AND 76;

**AMED Search Strategy: (27^th^ February 2015)**

1 exp probiotic agent/

2 "probio*".ti,ab.

3 "synbio*".ti,ab.

4 expsynbiotic agent/

5 acidophilus.ti,ab.

6 delbruecki.ti,ab.

7 johnsonii.ti,ab.

8 plantarum.ti,ab.

9 fermentum.ti,ab.

10 reuteri.ti,ab.

11 rhamnosus.ti,ab.

12 salivarius.ti,ab.

13 bifidum.ti,ab.

14 "bifidobacteri*".ti,ab.

15 exp bifidobacterium/ or bifidobacteriaceae/

16 saccharomycetales/ or exp saccharomyces/

17 "saccharomyc*".ti,ab.

18 enterococcaceae/ or exp enterococcus/

19 "enterococc*".ti,ab.

20 "dairy product* ".ti,ab.

21 exp yoghurt/

22 "yoghurt*".ti,ab.

23 "fermented product* ".ti,ab.

24 kefir.ti,ab.

25 bacillales/

26 lactobacillales/ or exp lactobacillus/ or lactobacillaceae/

27 "lactobacill*".ti,ab.

28 (casei or paracasei).ti,ab.

29 shirota.ti,ab.

30 ("Lactobac* casei Shirota" or LcS).ti,ab.

31 (lactobac* adj3 shirota).mp. [mp=abstract, heading words, title]

32 amylovorus.ti,ab.

33 bifidus.ti,ab.

34 brevis.ti,ab.

35 buchneri.ti,ab.

36 bulgaricus.ti,ab.

37 crispatus.ti,ab.

38 curvatus.ti,ab.

39 delbrueckii.ti,ab.

40 gasseri.ti,ab.

41 sakei.ti,ab.

42 pentosus.ti,ab.

43 helveticus.ti,ab.

44 "streptococc*".ti,ab.

45 streptococcaceae/ or exp streptococcus/

46 1 or 2 or 3 or 4 or 5 or 6 or 7 or 8 or 9 or 10 or 11 or 12 or 13 or 14 or 15 or 16 or 17 or 18 or 19 or 20 or 21 or 22 or 23 or 24 or 25 or 26 or 27 or 28 or 29 or 30 or 31 or 32 or 33 or 34 or 35 or 36 or 37 or 38 or 39 or 40 or 41 or 42 or 43 or 44 or 45

47 exp spinal cord injury/

48 exp cervical spine injury/

49 exp spine injury/

50 exp cervical spinal cord injury/

51 SCI.ti,ab.

52 ((spine or spinal or cervical or thoracic or lumbar or sacral) adj3 (trauma* or injur* or contus* or lacerat* or transection* or lesion* or wound* or broken or break* or fractur* or damag*)).mp. [mp=abstract, heading words, title]

53 ("central cord" adj3 (trauma* or injur* or contus* or lacerat* or transection* or lesion* or wound* or broken or break* or fractur* or damag*)).mp. [mp=abstract, heading words, title]

54 exp paraplegia/

55 (parapleg* or paraparesis).ti,ab.

56 exp quadriplegia/

57 (quadripleg* or quadriparesis).ti,ab.

58 (quadrapleg* or quadraparesis).ti,ab.

59 (tetrapleg* or tetraparesis).ti,ab.

60 ("spinal cord" adj3 patient*).mp. [mp=abstract, heading words, title]

61 (spine or spinal).jx.

62 47 or 48 or 49 or 50 or 51 or 52 or 53 or 54 or 55 or 56 or 57 or 58 or 59 or 60 or 61 (8766)

63 46 and 62

64 exp diarrhea/

65 exp Clostridium difficile/

66 clostridium difficile/ or exp clostridium/ or exp clostridium difficile infection/

67 exp pseudomembranous colitis/

68 "diarrh*ea".ti,ab.

69 (AAD or CDAD).ti,ab.

70 "c*diff* ".ti,ab.

71 (clostridium adj2 diff*).mp. [mp=abstract, heading words, title]

72 exp gastroenteritis/

73 "gastroenter*".ti,ab.

74 [exp antibiotic agent/ae [Adverse Drug Reaction]]

75 64 or 65 or 66 or 67 or 68 or 69 or 70 or 71 or 72 or 73 or 74

76 63 and 75

77 exp Microorganisms/ or exp Dietary Supplements/

78 46 or 77

79 exp Probiotics/

80 46 or 79

81 62 and 75 and 80

**PsycINFO Search Strategy: 27^th^ February 2015**

1 exp probiotic agent/

2 "probio*".ti,ab.

3 "synbio*".ti,ab.

4 expsynbiotic agent/

5 acidophilus.ti,ab.

6 delbruecki.ti,ab.

7 johnsonii.ti,ab.

8 plantarum.ti,ab.

9 fermentum.ti,ab.

10 reuteri.ti,ab.

11 rhamnosus.ti,ab.

12 salivarius.ti,ab.

13 bifidum.ti,ab.

14 "bifidobacteri*".ti,ab.

15 expbifidobacterium/ or bifidobacteriaceae/

16 saccharomycetales/ or exp saccharomyces/

17 "saccharomyc*".ti,ab.

18 enterococcaceae/ or exp enterococcus/

19 "enterococc*".ti,ab.

20 "dairy product* ".ti,ab.

21 exp yoghurt/

22 "yoghurt*".ti,ab.

23 "fermented product* ".ti,ab.

24 kefir.ti,ab.

25 bacillales/

26 lactobacillales/ or exp lactobacillus/ or lactobacillaceae/

27 "lactobacill*".ti,ab.

28 (casei or paracasei).ti,ab.

29 shirota.ti,ab.

30 ("Lactobac* casei Shirota" or LcS).ti,ab.

31 (lactobac* adj3 shirota).mp. [mp=title, abstract, heading word, table of contents, key concepts, original title, tests & measures]

32 amylovorus.ti,ab.

33 bifidus.ti,ab.

34 brevis.ti,ab.

35 buchneri.ti,ab.

36 bulgaricus.ti,ab.

37 crispatus.ti,ab.

38 curvatus.ti,ab.

39 delbrueckii.ti,ab.

40 gasseri.ti,ab.

41 sakei.ti,ab.

42 pentosus.ti,ab.

43 helveticus.ti,ab.

44 "streptococc*".ti,ab.

45 streptococcaceae/ or exp streptococcus/

46 1 or 2 or 3 or 4 or 5 or 6 or 7 or 8 or 9 or 10 or 11 or 12 or 13 or 14 or 15 or 16 or 17 or 18 or 19 or 20 or 21 or 22 or 23 or 24 or 25 or 26 or 27 or 28 or 29 or 30 or 31 or 32 or 33 or 34 or 35 or 36 or 37 or 38 or 39 or 40 or 41 or 42 or 43 or 44 or 45

47 exp spinal cord injury/

48 exp cervical spine injury/

49 exp spine injury/

50 exp cervical spinal cord injury/

51 SCI.ti,ab.

52 ((spine or spinal or cervical or thoracic or lumbar or sacral) adj3 (trauma* or injur* or contus* or lacerat* or transection* or lesion* or wound* or broken or break* or fractur* or damag*)).mp. [mp=title, abstract, heading word, table of contents, key concepts, original title, tests & measures]

53 ("central cord" adj3 (trauma* or injur* or contus* or lacerat* or transection* or lesion* or wound* or broken or break* or fractur* or damag*)).mp. [mp=title, abstract, heading word, table of contents, key concepts, original title, tests & measures]

54 exp paraplegia/

55 (parapleg* or paraparesis).ti,ab.

56 exp quadriplegia/

57 (quadripleg* or quadriparesis).ti,ab.

58 (quadrapleg* or quadraparesis).ti,ab.

59 (tetrapleg* or tetraparesis).ti,ab.

60 ("spinal cord" adj3 patient*).mp. [mp=title, abstract, heading word, table of contents, key concepts, original title, tests & measures]

61 (spine or spinal).jx.

62 47 or 48 or 49 or 50 or 51 or 52 or 53 or 54 or 55 or 56 or 57 or 58 or 59 or 60 or 61 (8875)

63 46 and 62

64 exp diarrhea/

65 exp Clostridium difficile/

66 clostridium difficile/ or exp clostridium/ or exp clostridium difficile infection/

67 exp pseudomembranous colitis/

68 "diarrh*ea".ti,ab.

69 (AAD or CDAD).ti,ab.

70 "c*diff* ".ti,ab.

71 (clostridium adj2 diff*).mp. [mp=title, abstract, heading word, table of contents, key concepts, original title, tests & measures]

72 exp gastroenteritis/

73 "gastroenter*".ti,ab.

74 [exp antibiotic agent/ae [Adverse Drug Reaction]]

75 64 or 65 or 66 or 67 or 68 or 69 or 70 or 71 or 72 or 73 or 74

76 63 and 75

77 exp Antibiotics/

78 exp Microorganisms/ or exp Dietary Supplements/

79 46 or 78

80 75 or 77

81 62 and 79 and 80

**Cochrane Search Strategy April 2015**

#1 MeSH descriptor: [Spinal Cord Injuries] explode all trees 914

#2 "spinal cord" 3139

#3 "spinal injur*" 324

#4 SCI 2911

#5 MeSH descriptor: [Paraplegia] explode all trees 166

#6 MeSH descriptor: [Quadriplegia] explode all trees 135

#7 parapleg* or tetrapleg* or quadrapleg* 609

#8 "spinal trauma" 25

#9 #1 or #2 or #3 or #4 or #5 or #6 or #7 or #8 5898

#10 MeSH descriptor: [Probiotics] explode all trees 1219

#11 MeSH descriptor: [Synbiotics] explode all trees 41

#12 MeSH descriptor: [Lactobacillaceae] explode all trees 1058

#13 MeSH descriptor: [Saccharomycetales] explode all trees 127

#14 MeSH descriptor: [Streptococcaceae] explode all trees 1542

#15 probio* or synbio* or lactobacil* or bifidobact* or saccharomy* or bifidobac* or microecological or enterococ* or streptococ* or casei or shirota or yoghurt 7784

#16 MeSH descriptor: [Yogurt] explode all trees 242

#17 #10 or #11 or #12 or #13 or #14 or #15 or #16 7867

#18 #9 and #17 61

#19 MeSH descriptor: [Diarrhea] explode all trees 2559

#20 diarrh*ea* 13815

#21 MeSH descriptor: [Clostridium difficile] explode all trees 171

#22 "c diff*" 339

#23 MeSH descriptor: [Gastroenteritis] explode all trees 4838

#24 AAD or CDAD 293

#25 MeSH descriptor: [Anti-Bacterial Agents] explode all trees 9374

#26 #19 or #20 or #21 or #22 or #23 or #24 or #25 27077

#27 #18 and #26 33

**Centre for Reviews and Dissemination**

Title search for **spinal cord injuries**

Any field search for **spinal cord**or **spinal injuries**  and**probiotics**

Any field search **spinal cord** or **spinal injuries** and **lactobacillus**

Any field search **spinal cord** or **spinal injuries** and **casei**

Any field search **spinal cord** or **spinal injuries** and **shirota**

Any field search **spinal cord** or **spinal injuries**and **clostridium**

**International Clinical Trials Registry**

**Spinal cord** and **diarrhea**

**Spinal injuries** and **diarrhea**

**Spinal cord or spinal injuries** and **lactobacillus**

**Spinal cord or spinal injuries** and **casei**

**Spinal cord or spinal injuries and shirota**

**Spinal cord** or **spinal injuries** and **clostridium**
